# Supplementary material for: A systems biology approach to explore the impact of maple tree dormancy release on sap variation and maple syrup quality
Source: Sci Rep. 2018 Oct 2;8:14658. doi: 10.1038/s41598-018-32940-y (PMC6168607; doi:10.1038/s41598-018-32940-y)
Supplement: Supplementary file 1 — Supplementary figures and legends [file 41598_2018_32940_MOESM1_ESM.pdf]

A systems biology approach to explore the impact of maple tree dormancy release on sap variation and maple syrup quality

Guillaume Quang Nguyen, Nathalie Martin, Mani Jain, Luc Lagacé, Christian R Landry, Marie Filteau

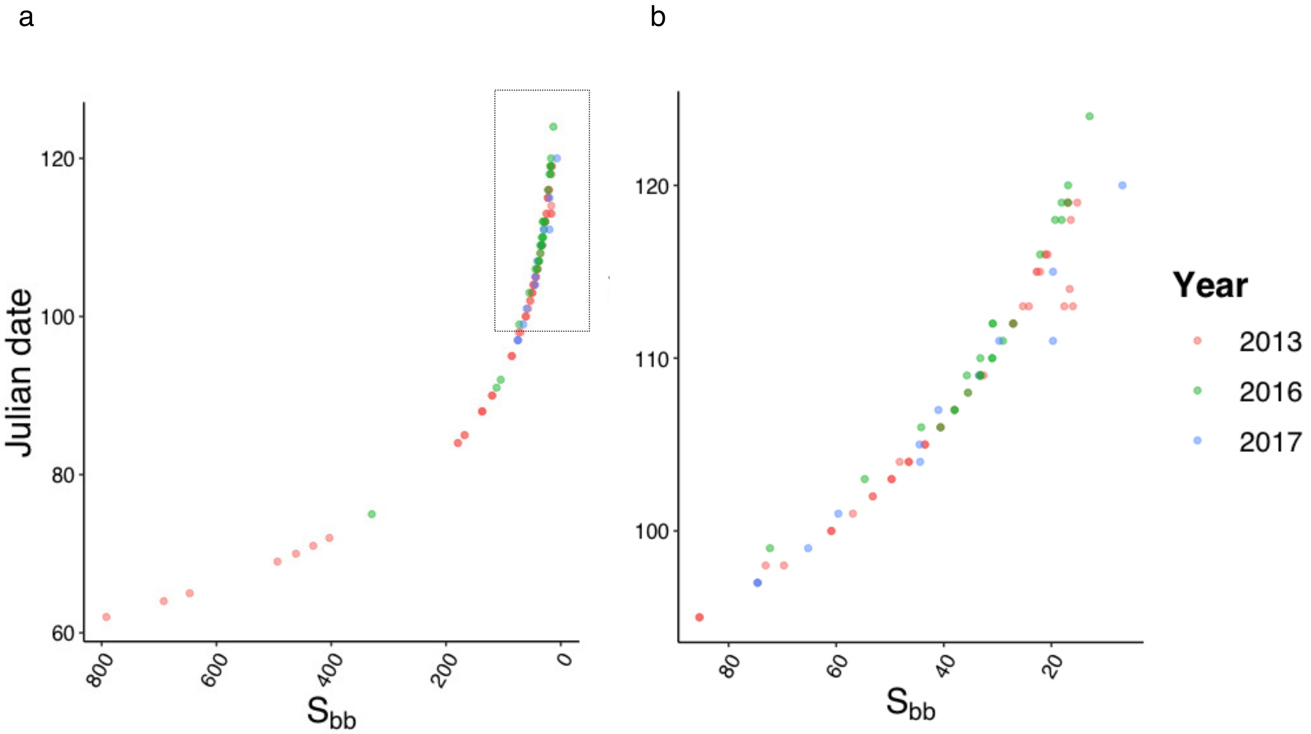

Supplementary Figure 1. Correlation between the Julian date (JD) and the Sbb index. a) Sbb index value for sample harvested in 2013 2016 and 2017. b) Sbb Axis change from 100 to 10 to show differences between years is observable starting from April (JD = 92 / Sbb ≈ 100).

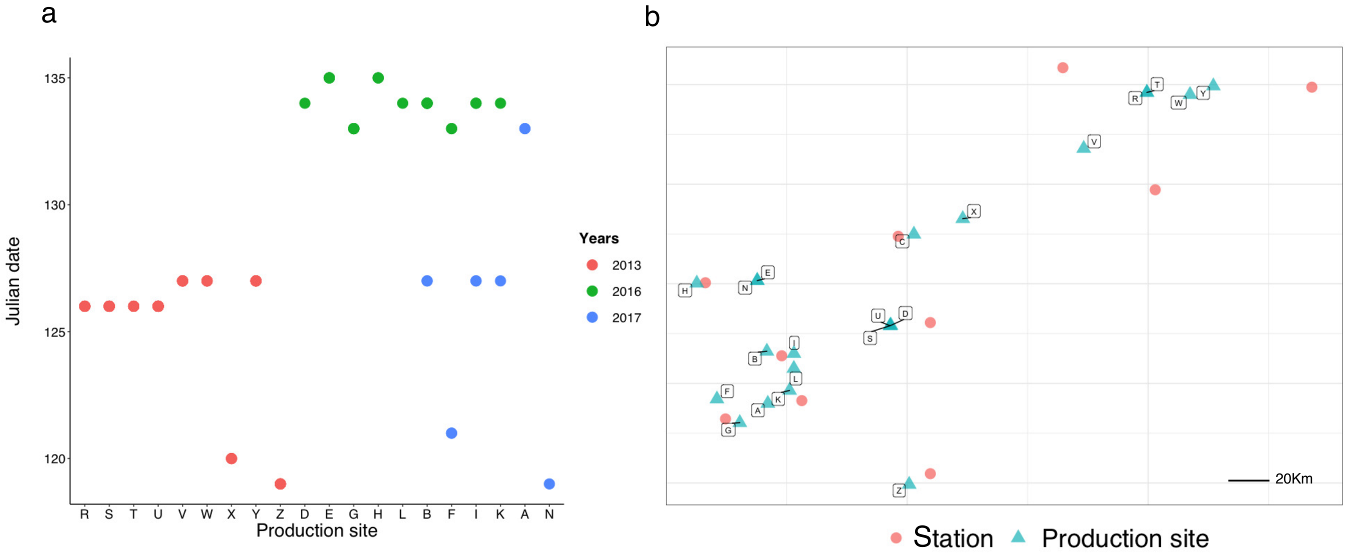

Supplementary Figure 2. Production site and weather station localization used to record the temperature for the Sbb model. a) The predicted Bud break date displayed by production site and year. The bud break date for the production sites vary according to years with a Julian date range of 119 to 135 (29/04 to 14/05). b) Schematic map of distance between the production sites and the weather station.

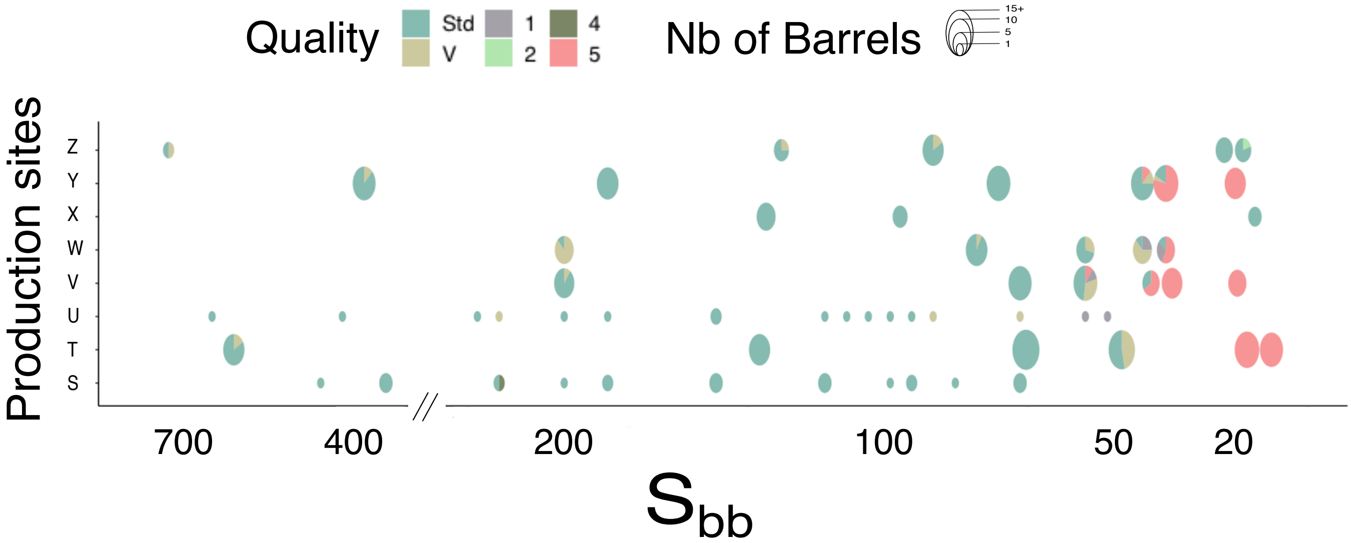

Supplementary Figure 3. Quality classification of the syrup barrels produced in 2013. Pie chart represents the proportion of syrup barrel quality according to the total number of barrels produced during the day. Pie charts are displayed by production site along the Sbb index axis. A change in quality from standard to class 5 is observable for most of the production sites after Sbb ≈20. "R" production site is not shown due to missing information on the defect classification.

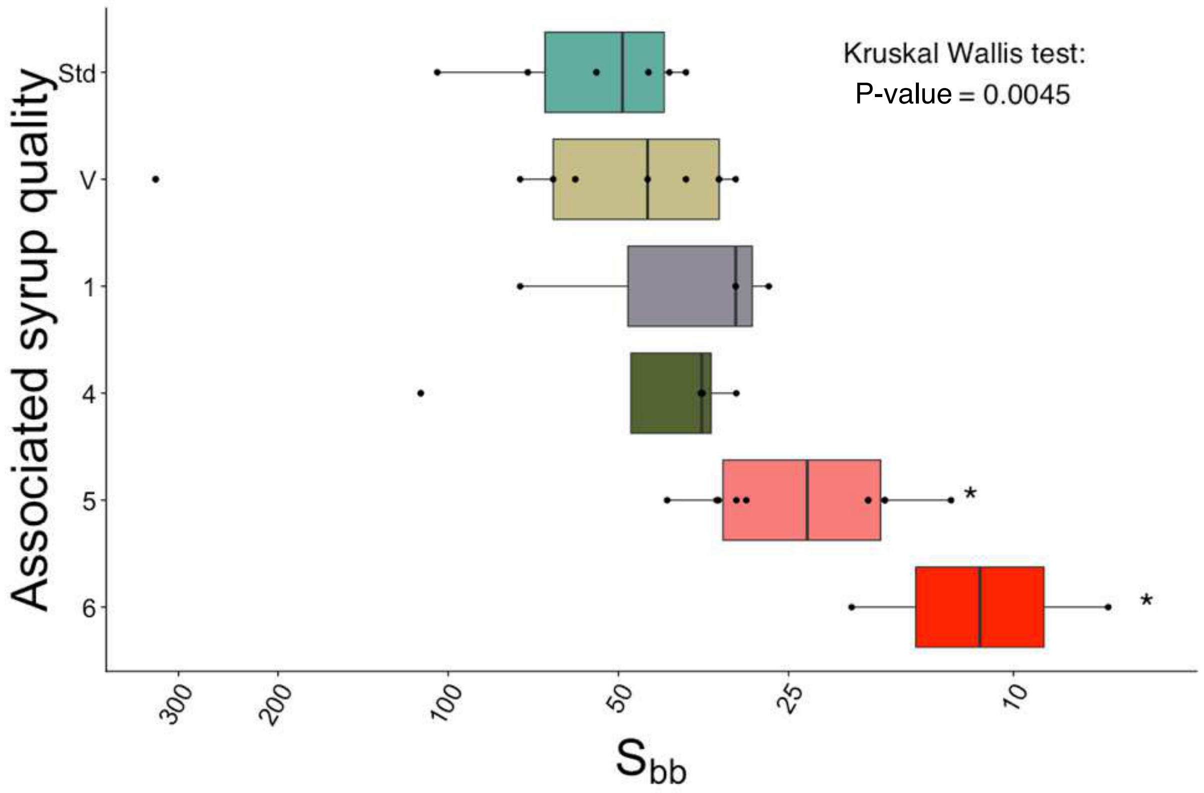

Supplementary Figure 4. Boxplots representing the sap quality samples from 2016 and 2017 harvesting period displayed by Sbb. Significant difference is observed between Std - class 5 and Std - class 6 (Dunns test$Pvalue < 0.05$). Boxplots show the median, 1st and 3rd quantiles.

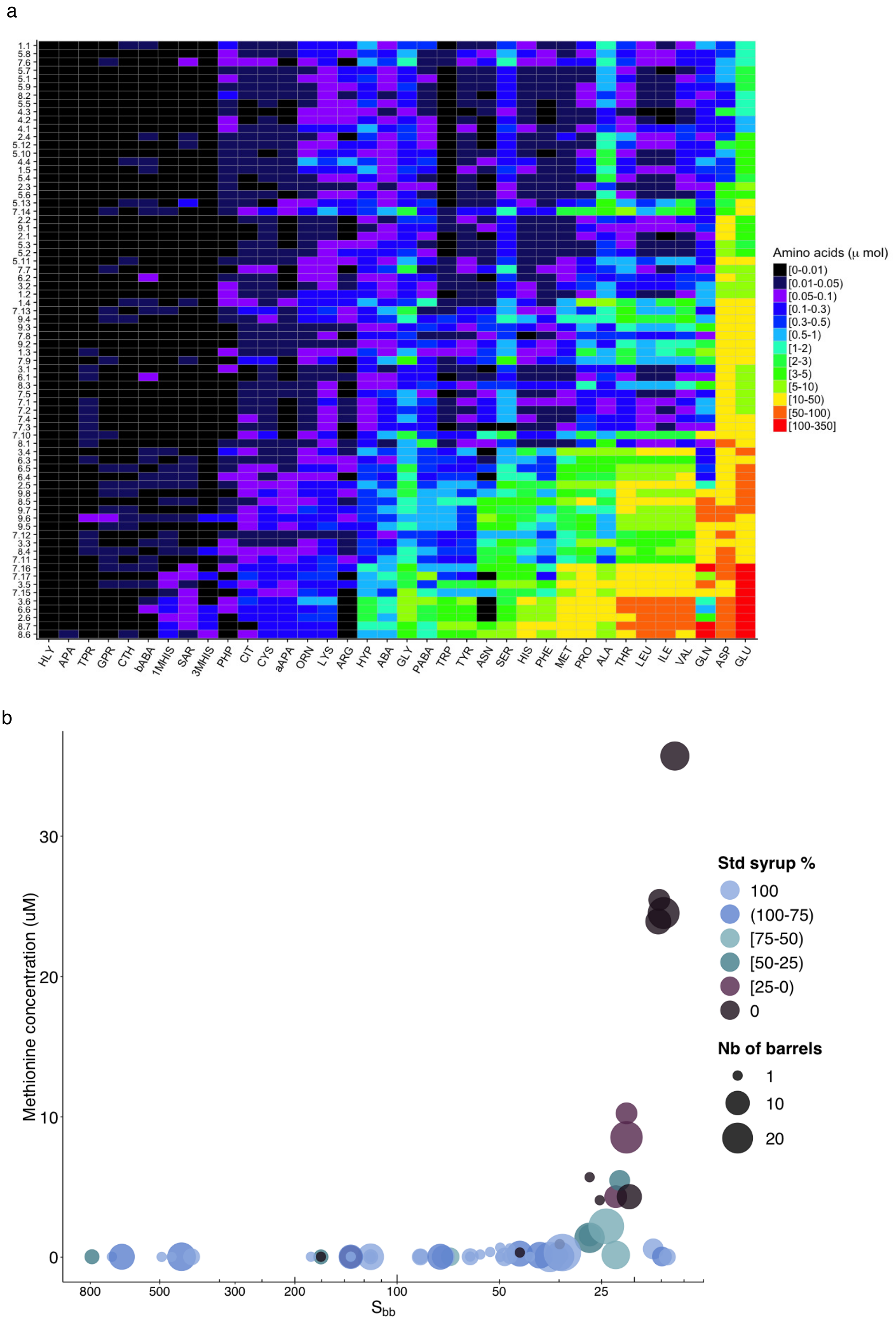

Supplementary Figure 5. Amino acids concentration for 2° brix sap samples harvested in 2013. a) Heatmap of quantified amino acid contents in sap samples. The y-axis displays samples ordered by increasing glutamate concentration. The color scale represents the amino acid concentration in μM. b) Relationship between methionine, Sbb and quality. Standard syrups including barrels produced at Sbb<41 contain very low amount of methionine.

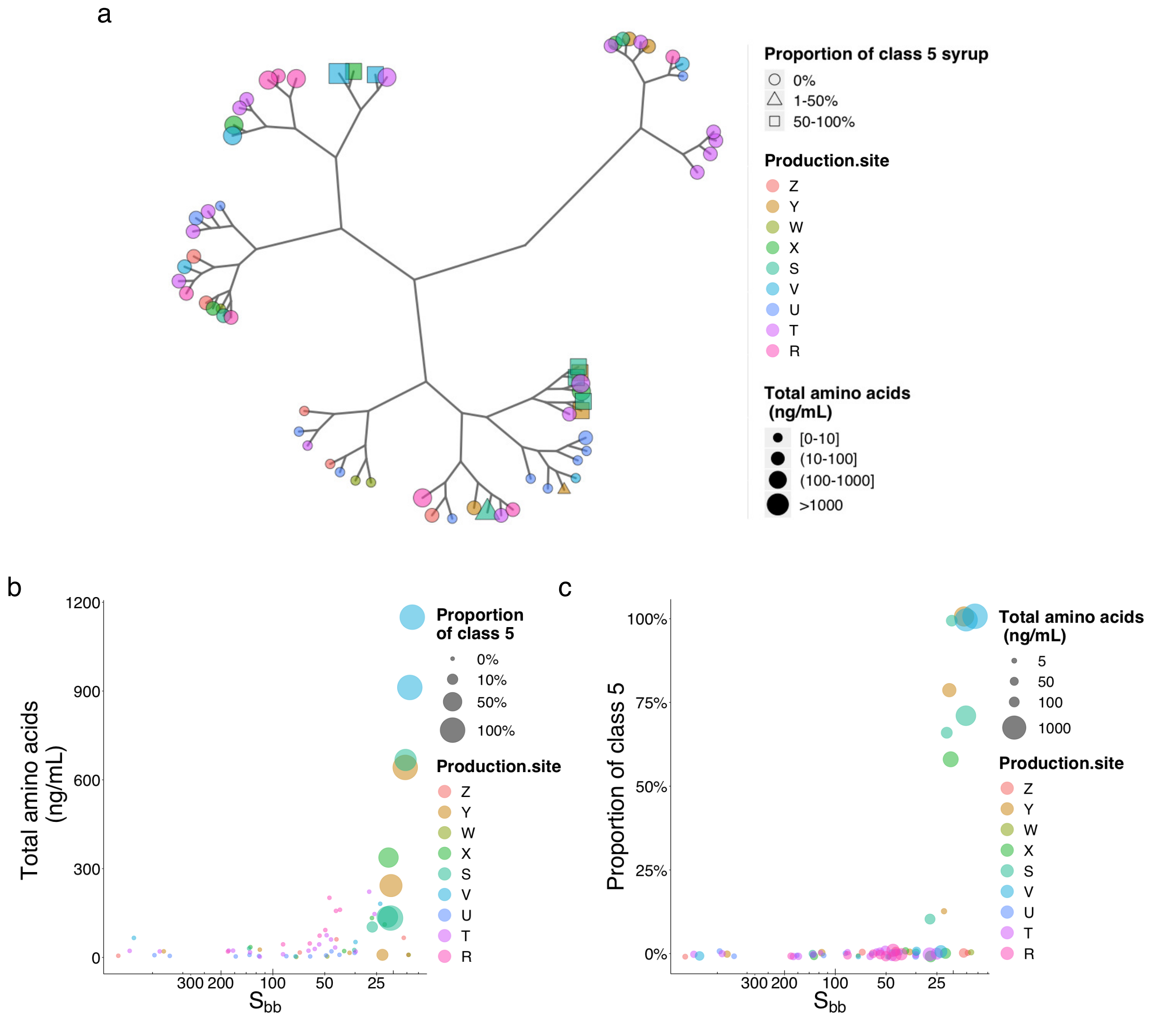

Supplementary Figure 6. Maple sap amino acid profiles do not vary by production sites. a) Unrooted tree (Ward's method) of amino acid profiles of 2013 samples showing unsupervised clustering. Branch length represents square root distances. Color and shape size represent the production sites and total amino acid content, respectively. Shapes reflect the proportion of class 5 syrup produced on the day of sampling. b) Class 5 syrup occurrence increases towards late Spring Dot size reflects the total amino acid concentration c) Total amino acid contents increase towards late Spring. Dot size reflects class 5 syrup proportion.

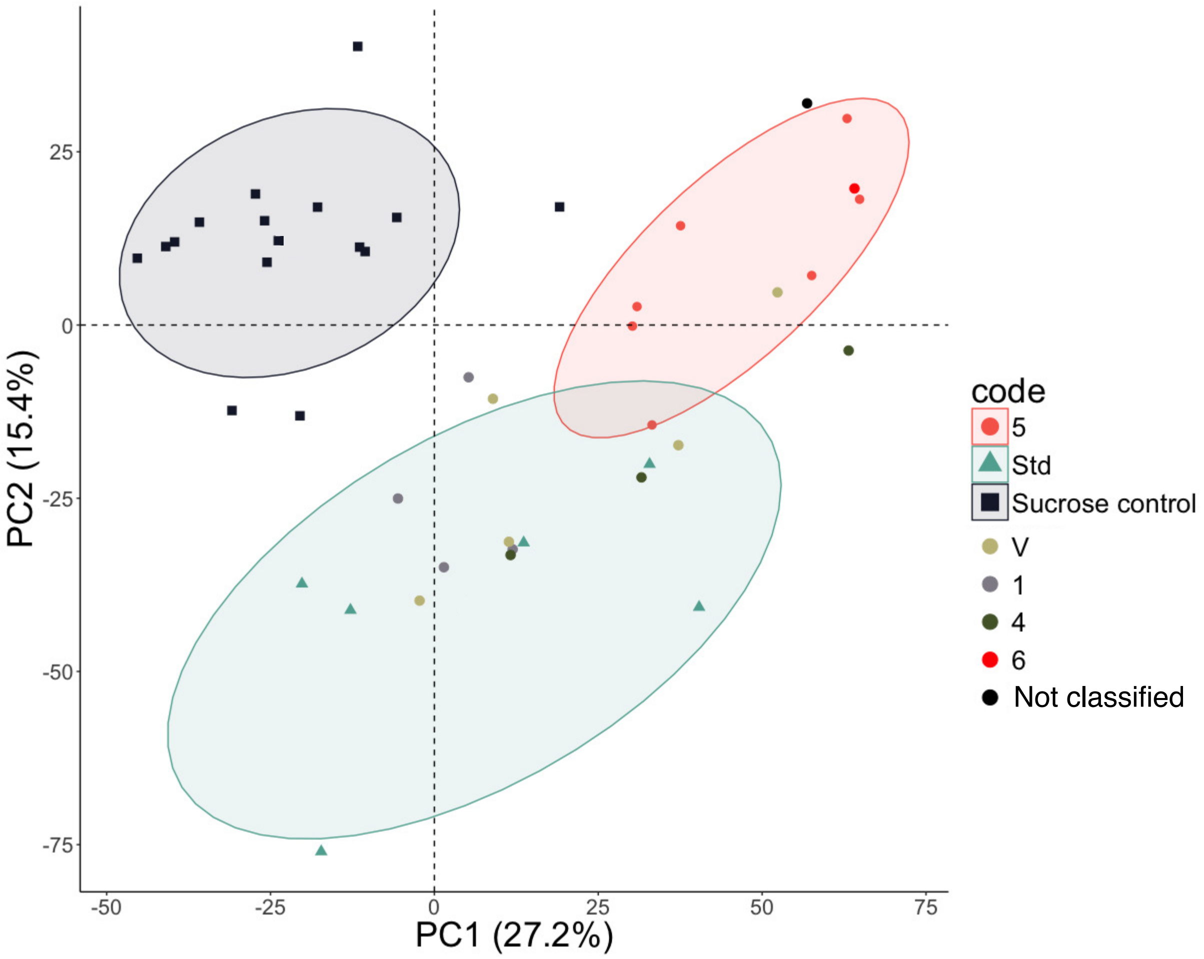

Supplementary Figure 7. Principal component analysis displaying the sap sample based on the mutant strain's fitness from the prototrophic collection. The yeast deletion collection presents a specific fitness profile depending on the sap quality. Std, class 5 and control media are highlighted by dispersal ellipses encompassing 70% of samples.

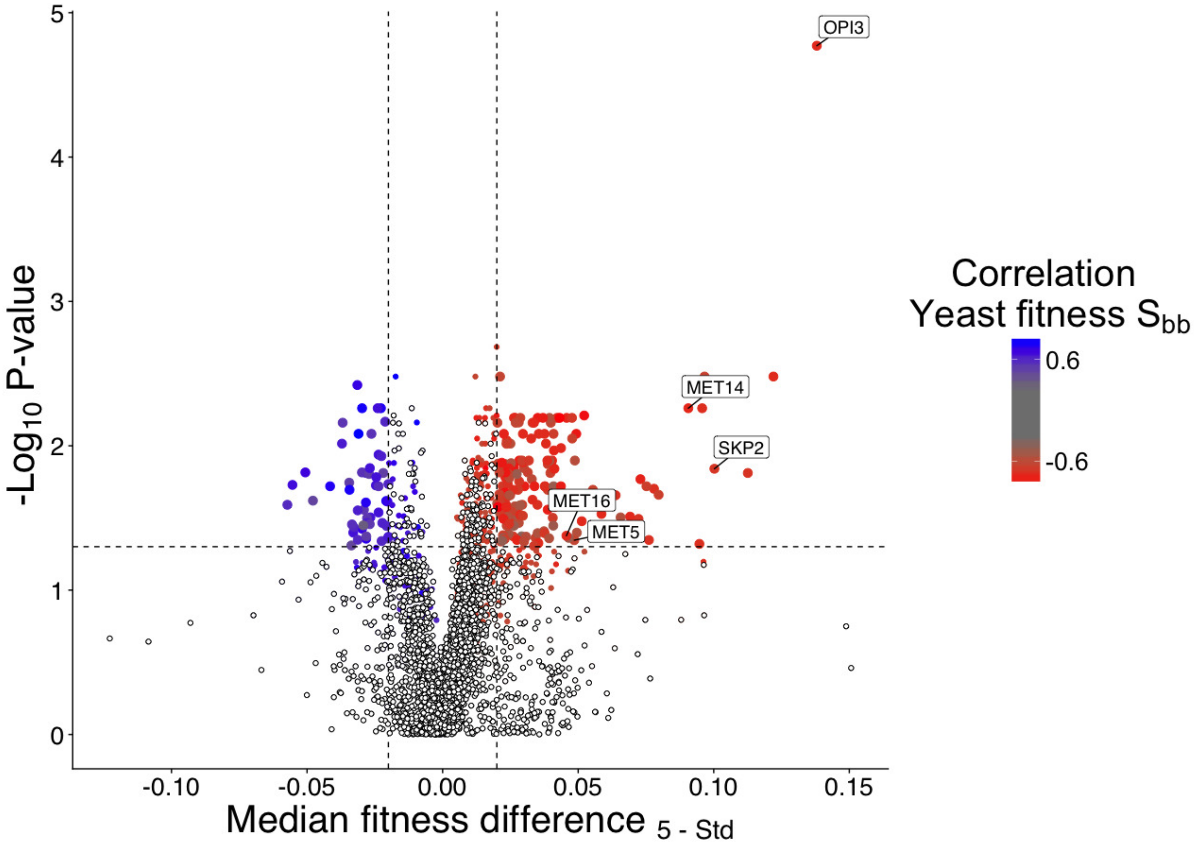

Supplementary Figure 8. Volcano plot of yeast fitness difference between sap quality (Std vs class 5) with overlaid correlation with Sbb as shown by the color scale. For the quality comparison, 218 significant strains were identified by a T-test comparison (adjusted $P-value < 0.01$ & mean fitness difference $+/-0.02$). Further, 1035 strains were identified as significatively correlated to the Sbb index (Spearman correlation adjusted $P-value < 0.01$), with 204 strains shared between the two lists. Dots size and color discriminates significant strains for the quality comparison and Sbb correlation, respectively. Strains involved in sulfur assimilation are labelled.

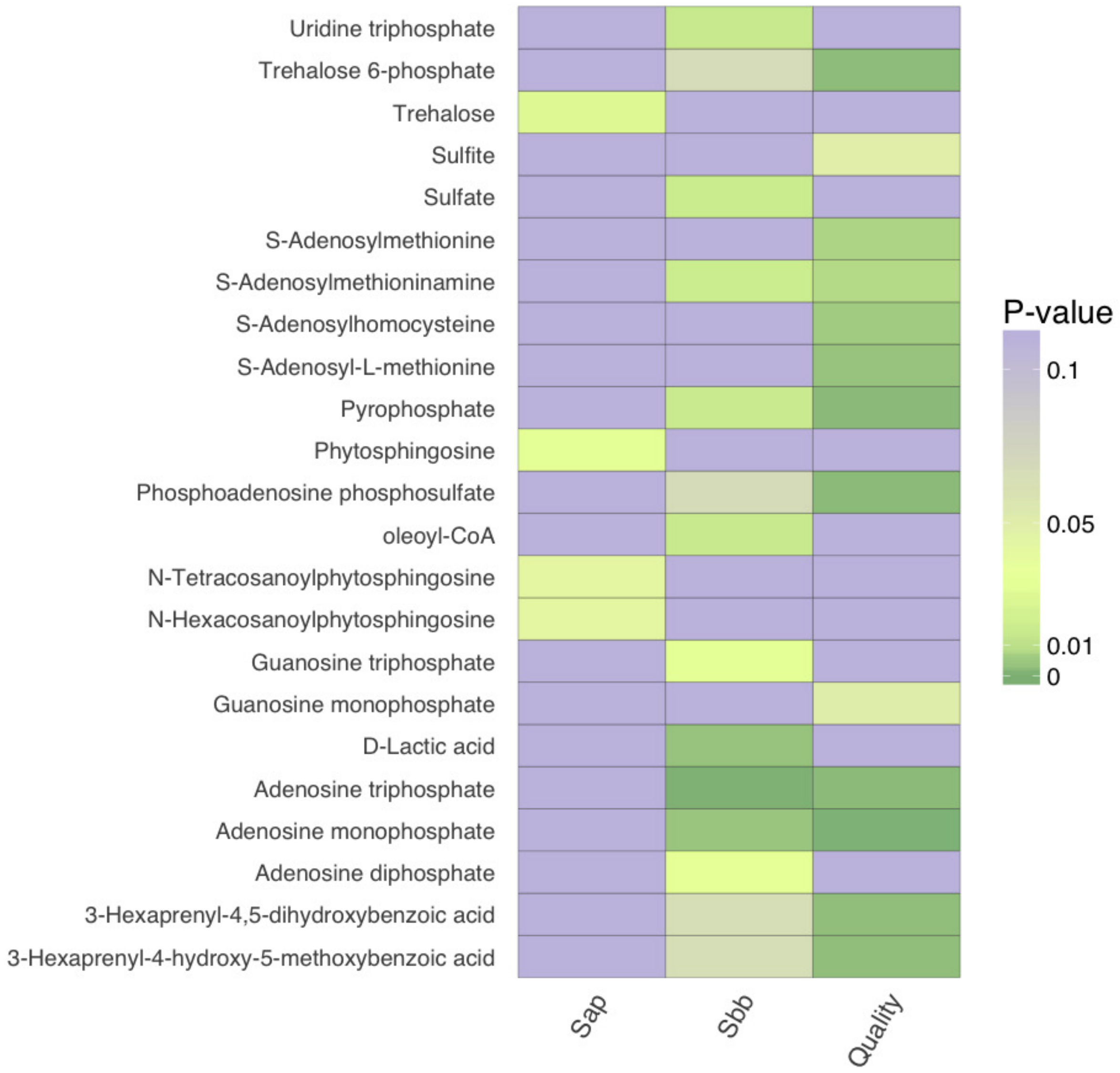

Supplementary Figure 9. Heatmap of enriched metabolites associated with genes in maple sap, Quality and Sbb lists ($P - value < 0.01$). Gene-metabolites associations were retrieved from YMDB.
